# Night work and sustainable working life - a prospective trajectory analysis of Swedish Twins

**DOI:** 10.1093/eurpub/ckac131.264

**Published:** 2022-10-25

**Authors:** A Ropponen, M Wang, A Raza, J Narusyte

**Affiliations:** 1 Finnish Institute of Occupational Health, Työterveyslaitos, Finland; 2 Division of Insurance Medicine, Department of Clinical Neuroscience, Karolinska Institutet, Stockholm, Sweden; 3 Center of Epidemiology and Community Medicine, Stockholm County Council, Stockholm, Sweden

## Abstract

**Objectives:**

Night work has been widely studied for the associations with diseases, sickness absence (SA) and disability pension (DP), but less for sustainable working life. We aimed to investigate the longitudinal changes in sustainable working life among those with or without baseline night work.

**Methods:**

Using data from Swedish national registers, sustainable working life was defined as employment during follow-up without interruptions due to SA/DP, unemployment, and old-age pension. Survey data for two cohorts (i.e., born before or after 1959) were utilized to assess night work at baseline (yes/no) in 1998-2003 and 2004-2006, respectively. The final samples for the two cohorts were 34680 and 19637, respectively. Group-based trajectory models were applied.

**Results:**

Among those born before 1959 (mean age 59 years, 13 years follow-up), a five-trajectory solution was best for those with and without night work. The trajectory groups were stable sustainable working life (38-42%), stable unsustainable working life (24-25%), early (13%) or later (13%) decreasing sustainable working life, and between sustainable and unsustainable working life (7%). Among those born after 1958 (mean age 37 years, follow-up 10 years), four trajectories were detected for those with night work: stable sustainable working life (81%), stable unsustainable working life (6%) and increasing (7%) and decreasing (5%) sustainable working life. For those without nightwork, a three-trajectory solution was best: stable sustainable working life (83%), stable unsustainable working life (6%) and between sustainable and unsustainable working life (11%).

**Conclusions:**

Sustainable working life was similar across baseline night work statuses of older cohort but differed in younger cohort. The findings suggest that at least night work at one time point does not affect sustainable working life. However, night work at early stages of working life could be accompanied with lifestyle counselling for sleep and recovery.

**Key messages:**

• Trajectories of sustainable working life seem similar for those with or without night work adding to the understanding of effects of such work to health.

• From public health perspective, counselling employees with night work already at early stages of working life for health behaviours including sleep and recovery could support sustainable working life.

